# A joint model for nonparametric functional mapping of longitudinal trajectory and time-to-event

**DOI:** 10.1186/1471-2105-7-138

**Published:** 2006-03-15

**Authors:** Min Lin, Rongling Wu

**Affiliations:** 1 Department of Statistics, University of Florida, Gainesville, FL 32611, USA; 2 Department of Biostatistics and Bioinformatics, Duke University, Durham, North Carolina 27710, USA

## Abstract

**Background:**

The characterization of the relationship between a longitudinal response process and a time-to-event has been a pressing challenge in biostatistical research. This has emerged as an important issue in genetic studies when one attempts to detect the common genes or quantitative trait loci (QTL) that govern both a longitudinal trajectory and developmental event.

**Results:**

We present a joint statistical model for functional mapping of dynamic traits in which the event times and longitudinal traits are taken to depend on a common set of genetic mechanisms. By fitting the Legendre polynomial of orthogonal properties for the time-dependent mean vector, our model does not rely on any curve, which is different from earlier parametric models of functional mapping. This newly developed nonparametric model is demonstrated and validated by an example for a forest tree in which stemwood growth and the time to first flower are jointly modelled.

**Conclusion:**

Our model allows for the detection of specific QTL that govern both longitudinal traits and developmental processes through either pleiotropic effects or close linkage, or both. This model will have great implications for integrating longitudinal and event data to gain better insights into comprehensive biology and biomedicine.

## Background

Although there has been a upsurge of interest in jointly modelling longitudinal and event data during the last decade [1-9], no statistical models have been developed to characterize the shared genetic basis for these two types of traits. In biomedicine, the identification of specific genetic variants responsible for an HIV patient's time-dependent CD4 count and for the time to onset of AIDS symptoms can help to design individualized drugs to control this patient's progression to AIDS. Similarly, in studies of prostate cancer, a shared genetic basis between prostate specific antigen, repeatedly measured for patients following treatment for prostate cancer, and the time to disease recurrence can be used to make optimal treatment schedules for patients. In plants, knowledge about whether the genetic loci for reproductive behaviors, such as the time to first flower and the time to form seeds, also govern growth rates and sizes of plants helps to understand the etiology of plant's adaptation to the environment in which they are grown.

The genetic mapping of quantitative trait loci (QTL) that are responsible for longitudinal traits has long been a difficult issue because of the dynamic features of these traits. More recently, part of this difficulty has been solved by integrating the statistical analysis of longitudinal data into a QTL mapping framework, leading to a so-called *functional mapping *strategy [10-16]. Statistical models for functional mapping were established on the belief that biological processes can be described by mathematical functions. One of the most significant examples for this is the use of S-shaped logistic curves to model growth trajectories. West et al. [17] indicated from fundamental principles of biophysical processes that logistic forms of growth are biologically crucial for the maintenance of optimal metabolic level and, thereby, the best use of available resources for an organism from birth to adulthood. Because of the embedment of fundamental biological principles within the modelling model, functional mapping provides a quantitative framework for testing biologically relevant hypotheses at the interplay between gene actions and development.

The concept of functional mapping can be further extended to jointly mapping a longitudinal variable and a time-to-event by incorporating statistical theories developed to characterize the relationships between longitudinal response and event processes [1-9]. However, original functional mapping models for a dynamic trait reply upon explicit mathematical functions that describe the development of the trait. In practice, there are also many situations in which no appropriate curves can be used to describe a biological process. To model an arbitrary shape of curves, a different statistical model based on nonparametric theory should be formulated. Polynomial analyses that can be specified by varying orders have power to fit curves with arbitrary shapes. As shown by Kirkpatrick and Heckman [18], Legendre polynomials have several favorable properties for curve fitting which include: (1) the functions are orthogonal, (2) it is flexible to fit sparse data, (3) higher orders are estimable for high levels of curve complexity and (4) computation is fast because of good convergence.

The purpose of this article is to develop a joint statistical model for nonparametric functional mapping of longitudinal trajectories based on the Legendre polynomials, integrated with time-to-events. This joint model is constructed within the maximum likelihood context, including simultaneously modelling of the mean vector (based on nonparametric approaches) and covariance matrix (based on parametric approaches). By analyzing stem volume growth data in an example of a forest tree, we will demonstrates the implications of our joint model. Lastly, the advantages of our model in general biomedical and biological research and the areas in which the model can be further refined are discussed.

## The Model

### The likelihood function

Consider a mapping population of size *n *for which a number of molecular markers are genotyped, aimed to identify QTL for a longitudinal trait and time-to-event. Every individual of the mapping population is measured for the longitudinal trait at multiple (say *T*) time points (**y**) and a time-to-event (*z*). Variable *z *can be the time to first flower, the timing of cancer malignance, the time of mortality, or the events that happen at a time. The inference of unknown QTL genotypes for the phenotypic traits based on observed marker information (**M**) can be made due to co-segregation between the QTL and markers.

Suppose there are two segregating QTL for longitudinal and event traits in the mapping population, each with genotypes 2, 1 and 0. These two QTL are assumed to be linked or associated with and, therefore, can be inferred from, markers. The joint likelihood function of the two types of phenotypic data and marker information at the two underlying QTL is written as

L(Ω|y,z,M)=∏i=1n∑j1=02∑j2=02[ϖj1j2|ifj1j2(yi,zi;uj1j2,vj1j2,∑)],     (1)
 MathType@MTEF@5@5@+=feaafiart1ev1aaatCvAUfKttLearuWrP9MDH5MBPbIqV92AaeXatLxBI9gBaebbnrfifHhDYfgasaacH8akY=wiFfYdH8Gipec8Eeeu0xXdbba9frFj0=OqFfea0dXdd9vqai=hGuQ8kuc9pgc9s8qqaq=dirpe0xb9q8qiLsFr0=vr0=vr0dc8meaabaqaciaacaGaaeqabaqabeGadaaakeaacqWGmbatcqGGOaakiiqacqWFPoWvcqGG8baFieqacqGF5bqEcqGGSaalcqWG6bGEcqGGSaalcqGFnbqtcqGGPaqkcqGH9aqpdaqeWbqaamaaqahabaWaaabCaeaacqGGBbWwiiGacqqFwpGDdaWgaaWcbaGaemOAaO2aaSbaaWqaaiabigdaXaqabaWccqWGQbGAdaWgaaadbaGaeGOmaidabeaaliabcYha8jabdMgaPbqabaGccqWGMbGzdaWgaaWcbaGaemOAaO2aaSbaaWqaaiabigdaXaqabaWccqWGQbGAdaWgaaadbaGaeGOmaidabeaaaSqabaGccqGGOaakcqGF5bqEdaWgaaWcbaGaemyAaKgabeaakiabcYcaSiabdQha6naaBaaaleaacqWGPbqAaeqaaOGaei4oaSJae4xDau3aaSbaaSqaaiabdQgaQnaaBaaameaacqaIXaqmaeqaaSGaemOAaO2aaSbaaWqaaiabikdaYaqabaaaleqaaOGaeiilaWIaemODay3aaSbaaSqaaiabdQgaQnaaBaaameaacqaIXaqmaeqaaSGaemOAaO2aaSbaaWqaaiabikdaYaqabaaaleqaaOGaeiilaWIaeyyeIuUaeiykaKIaeiyxa0faleaacqWGQbGAdaWgaaadbaGaeGOmaidabeaaliabg2da9iabicdaWaqaaiabikdaYaqdcqGHris5aaWcbaGaemOAaO2aaSbaaWqaaiabigdaXaqabaWccqGH9aqpcqaIWaamaeaacqaIYaGma0GaeyyeIuoaaSqaaiabdMgaPjabg2da9iabigdaXaqaaiabd6gaUbqdcqGHpis1aOGaeiilaWIaaCzcaiaaxMaadaqadaqaaiabigdaXaGaayjkaiaawMcaaaaa@81DF@

where **Ω **is the unknown vector that defines the QTL positions, time-dependent QTL effects (uj1j2
 MathType@MTEF@5@5@+=feaafiart1ev1aaatCvAUfKttLearuWrP9MDH5MBPbIqV92AaeXatLxBI9gBaebbnrfifHhDYfgasaacH8akY=wiFfYdH8Gipec8Eeeu0xXdbba9frFj0=OqFfea0dXdd9vqai=hGuQ8kuc9pgc9s8qqaq=dirpe0xb9q8qiLsFr0=vr0=vr0dc8meaabaqaciaacaGaaeqabaqabeGadaaakeaaieqacqWF1bqDdaWgaaWcbaGaemOAaO2aaSbaaWqaaiabigdaXaqabaWccqWGQbGAdaWgaaadbaGaeGOmaidabeaaaSqabaaaaa@335D@ and vj1j2
 MathType@MTEF@5@5@+=feaafiart1ev1aaatCvAUfKttLearuWrP9MDH5MBPbIqV92AaeXatLxBI9gBaebbnrfifHhDYfgasaacH8akY=wiFfYdH8Gipec8Eeeu0xXdbba9frFj0=OqFfea0dXdd9vqai=hGuQ8kuc9pgc9s8qqaq=dirpe0xb9q8qiLsFr0=vr0=vr0dc8meaabaqaciaacaGaaeqabaqabeGadaaakeaacqWG2bGDdaWgaaWcbaGaemOAaO2aaSbaaWqaaiabigdaXaqabaWccqWGQbGAdaWgaaadbaGaeGOmaidabeaaaSqabaaaaa@3359@) and covariance matrix (Σ), ϖj1j2|i
 MathType@MTEF@5@5@+=feaafiart1ev1aaatCvAUfKttLearuWrP9MDH5MBPbIqV92AaeXatLxBI9gBaebbnrfifHhDYfgasaacH8akY=wiFfYdH8Gipec8Eeeu0xXdbba9frFj0=OqFfea0dXdd9vqai=hGuQ8kuc9pgc9s8qqaq=dirpe0xb9q8qiLsFr0=vr0=vr0dc8meaabaqaciaacaGaaeqabaqabeGadaaakeaaiiGacqWFwpGDdaWgaaWcbaGaemOAaO2aaSbaaWqaaiabigdaXaqabaWccqWGQbGAdaWgaaadbaGaeGOmaidabeaaliabcYha8jabdMgaPbqabaaaaa@369F@ is the mixture proportion expressed as the conditional probability of a joint genotype *j*_1_*j*_2 _for the longitudinal and event QTL given marker genotypes for individual *i *and fj1j2
 MathType@MTEF@5@5@+=feaafiart1ev1aaatCvAUfKttLearuWrP9MDH5MBPbIqV92AaeXatLxBI9gBaebbnrfifHhDYfgasaacH8akY=wiFfYdH8Gipec8Eeeu0xXdbba9frFj0=OqFfea0dXdd9vqai=hGuQ8kuc9pgc9s8qqaq=dirpe0xb9q8qiLsFr0=vr0=vr0dc8meaabaqaciaacaGaaeqabaqabeGadaaakeaacqWGMbGzdaWgaaWcbaGaemOAaO2aaSbaaWqaaiabigdaXaqabaWccqWGQbGAdaWgaaadbaGaeGOmaidabeaaaSqabaaaaa@3339@ is the (*T *+ 1)-dimensional multivariate normal distribution function with mean vector (uj1j2
 MathType@MTEF@5@5@+=feaafiart1ev1aaatCvAUfKttLearuWrP9MDH5MBPbIqV92AaeXatLxBI9gBaebbnrfifHhDYfgasaacH8akY=wiFfYdH8Gipec8Eeeu0xXdbba9frFj0=OqFfea0dXdd9vqai=hGuQ8kuc9pgc9s8qqaq=dirpe0xb9q8qiLsFr0=vr0=vr0dc8meaabaqaciaacaGaaeqabaqabeGadaaakeaaieqacqWF1bqDdaWgaaWcbaGaemOAaO2aaSbaaWqaaiabigdaXaqabaWccqWGQbGAdaWgaaadbaGaeGOmaidabeaaaSqabaaaaa@335D@, vj1j2
 MathType@MTEF@5@5@+=feaafiart1ev1aaatCvAUfKttLearuWrP9MDH5MBPbIqV92AaeXatLxBI9gBaebbnrfifHhDYfgasaacH8akY=wiFfYdH8Gipec8Eeeu0xXdbba9frFj0=OqFfea0dXdd9vqai=hGuQ8kuc9pgc9s8qqaq=dirpe0xb9q8qiLsFr0=vr0=vr0dc8meaabaqaciaacaGaaeqabaqabeGadaaakeaacqWG2bGDdaWgaaWcbaGaemOAaO2aaSbaaWqaaiabigdaXaqabaWccqWGQbGAdaWgaaadbaGaeGOmaidabeaaaSqabaaaaa@3359@) and covariance matrix Σ.

### Conditional probabilities

There are different descriptions of the conditional probability, depending on the type of the mapping population. If the mapping population is an experimental cross initiated with two contrasting parents, such as the F_2 _or backcross, the conditional probability is described in terms of the recombination fractions between the markers and QTL [12,19]. If the two QTL are bracketed by different pairs of markers, the conditional probability of joint QTL genotypes given the marker intervals can be expressed as the product of the corresponding conditional probabilities for QTL genotypes given a single marker interval. If the two QTL are located at the same marker interval, the conditional probabilities should be derived using the principle of 4-point analysis. For a natural population, the association between the QTL and markers can be described by the coefficients of linkage disequilibria [17]

### Modelling the mean vector

The choice of a mean function for a longitudinal trait is based on theory or past experience that suggests a certain mathematical form for the time-dependent mean. However, it would be essential to derive a general approach that can fit any kind of curves. By choosing different orders of orthogonal polynomials, the Legendre function has potential to approximate the functional relationships between trait values and times to any specified degree of precision. The Legendre polynomials are solutions to a very important differential equation, the Legendre equation,

(1−x2)d2zdx2−2xdzdx+r(r+1)z=0.
 MathType@MTEF@5@5@+=feaafiart1ev1aaatCvAUfKttLearuWrP9MDH5MBPbIqV92AaeXatLxBI9gBaebbnrfifHhDYfgasaacH8akY=wiFfYdH8Gipec8Eeeu0xXdbba9frFj0=OqFfea0dXdd9vqai=hGuQ8kuc9pgc9s8qqaq=dirpe0xb9q8qiLsFr0=vr0=vr0dc8meaabaqaciaacaGaaeqabaqabeGadaaakeaacqGGOaakcqaIXaqmcqGHsislcqWG4baEdaahaaWcbeqaaiabikdaYaaakiabcMcaPmaalaaabaGaemizaq2aaWbaaSqabeaacqaIYaGmaaGccqWG6bGEaeaacqWGKbazcqWG4baEdaahaaWcbeqaaiabikdaYaaaaaGccqGHsislcqaIYaGmcqWG4baEdaWcaaqaaiabdsgaKjabdQha6bqaaiabdsgaKjabdIha4baacqGHRaWkcqWGYbGCcqGGOaakcqWGYbGCcqGHRaWkcqaIXaqmcqGGPaqkcqWG6bGEcqGH9aqpcqaIWaamcqGGUaGlaaa@4F6C@

The polynomials may be denoted by *P*_*r*_(*x*), called the Legendre polynomial of order *r*. The polynomials are either even or odd functions of *x *for even or odd orders *r*.

The general form of a Legendre polynomial of order *k *is given by the sum,

Pr(x)=∑k=0K(−1)k(2r−2k)!2rk!(r−k)!(r−2k)!xr−2k,     (2)
 MathType@MTEF@5@5@+=feaafiart1ev1aaatCvAUfKttLearuWrP9MDH5MBPbIqV92AaeXatLxBI9gBaebbnrfifHhDYfgasaacH8akY=wiFfYdH8Gipec8Eeeu0xXdbba9frFj0=OqFfea0dXdd9vqai=hGuQ8kuc9pgc9s8qqaq=dirpe0xb9q8qiLsFr0=vr0=vr0dc8meaabaqaciaacaGaaeqabaqabeGadaaakeaacqWGqbaudaWgaaWcbaGaemOCaihabeaakiabcIcaOiabdIha4jabcMcaPiabg2da9maaqahabaGaeiikaGIaeyOeI0IaeGymaeJaeiykaKYaaWbaaSqabeaacqWGRbWAaaaabaGaem4AaSMaeyypa0JaeGimaadabaGaem4saSeaniabggHiLdGcdaWcaaqaaiabcIcaOiabikdaYiabdkhaYjabgkHiTiabikdaYiabdUgaRjabcMcaPiabcgcaHaqaaiabikdaYmaaCaaaleqabaGaemOCaihaaOGaem4AaSMaeiyiaeIaeiikaGIaemOCaiNaeyOeI0Iaem4AaSMaeiykaKIaeiyiaeIaeiikaGIaemOCaiNaeyOeI0IaeGOmaiJaem4AaSMaeiykaKIaeiyiaecaaiabdIha4naaCaaaleqabaGaemOCaiNaeyOeI0IaeGOmaiJaem4AaSgaaOGaeiilaWIaaCzcaiaaxMaadaqadaqaaiabikdaYaGaayjkaiaawMcaaaaa@64B5@

where *K *= *r*/2 or (*r *- 1)/2 whichever is an integer. This polynomial is defined over the interval [-1, 1]. From Eq. 5, we show the first few polynomials as

P0(x)=1P1(x)=xP2(x)=12(3x2−1)P3(x)=12(5x3−3x)P4(x)=18(35x4−30x2+3)P5(x)=18(63x5−70x3+15x)P6(x)=116(231x6−315x4+105x2−5).
 MathType@MTEF@5@5@+=feaafiart1ev1aaatCvAUfKttLearuWrP9MDH5MBPbIqV92AaeXatLxBI9gBaebbnrfifHhDYfgasaacH8akY=wiFfYdH8Gipec8Eeeu0xXdbba9frFj0=OqFfea0dXdd9vqai=hGuQ8kuc9pgc9s8qqaq=dirpe0xb9q8qiLsFr0=vr0=vr0dc8meaabaqaciaacaGaaeqabaqabeGadaaakqaabeqaaiabdcfaqnaaBaaaleaacqaIWaamaeqaaOGaeiikaGIaemiEaGNaeiykaKIaeyypa0JaeGymaedabaGaemiuaa1aaSbaaSqaaiabigdaXaqabaGccqGGOaakcqWG4baEcqGGPaqkcqGH9aqpcqWG4baEaeaacqWGqbaudaWgaaWcbaGaeGOmaidabeaakiabcIcaOiabdIha4jabcMcaPiabg2da9maalaaabaGaeGymaedabaGaeGOmaidaaiabcIcaOiabiodaZiabdIha4naaCaaaleqabaGaeGOmaidaaOGaeyOeI0IaeGymaeJaeiykaKcabaGaemiuaa1aaSbaaSqaaiabiodaZaqabaGccqGGOaakcqWG4baEcqGGPaqkcqGH9aqpdaWcaaqaaiabigdaXaqaaiabikdaYaaacqGGOaakcqaI1aqncqWG4baEdaahaaWcbeqaaiabiodaZaaakiabgkHiTiabiodaZiabdIha4jabcMcaPaqaaiabdcfaqnaaBaaaleaacqaI0aanaeqaaOGaeiikaGIaemiEaGNaeiykaKIaeyypa0ZaaSaaaeaacqaIXaqmaeaacqaI4aaoaaGaeiikaGIaeG4mamJaeGynauJaemiEaG3aaWbaaSqabeaacqaI0aanaaGccqGHsislcqaIZaWmcqaIWaamcqWG4baEdaahaaWcbeqaaiabikdaYaaakiabgUcaRiabiodaZiabcMcaPaqaaiabdcfaqnaaBaaaleaacqaI1aqnaeqaaOGaeiikaGIaemiEaGNaeiykaKIaeyypa0ZaaSaaaeaacqaIXaqmaeaacqaI4aaoaaGaeiikaGIaeGOnayJaeG4mamJaemiEaG3aaWbaaSqabeaacqaI1aqnaaGccqGHsislcqaI3aWncqaIWaamcqWG4baEdaahaaWcbeqaaiabiodaZaaakiabgUcaRiabigdaXiabiwda1iabdIha4jabcMcaPaqaaiabdcfaqnaaBaaaleaacqaI2aGnaeqaaOGaeiikaGIaemiEaGNaeiykaKIaeyypa0ZaaSaaaeaacqaIXaqmaeaacqaIXaqmcqaI2aGnaaGaeiikaGIaeGOmaiJaeG4mamJaeGymaeJaemiEaG3aaWbaaSqabeaacqaI2aGnaaGccqGHsislcqaIZaWmcqaIXaqmcqaI1aqncqWG4baEdaahaaWcbeqaaiabisda0aaakiabgUcaRiabigdaXiabicdaWiabiwda1iabdIha4naaCaaaleqabaGaeGOmaidaaOGaeyOeI0IaeGynauJaeiykaKIaeiOla4caaaa@ABA9@

In this modelling, independent variable *x *is expressed as time *t*, which is adjusted, to rescale the measurement times to the range of the orthogonal function [-1, 1], by

t*=−1+2(t−tmin⁡)tmax⁡−tmin⁡,
 MathType@MTEF@5@5@+=feaafiart1ev1aaatCvAUfKttLearuWrP9MDH5MBPbIqV92AaeXatLxBI9gBaebbnrfifHhDYfgasaacH8akY=wiFfYdH8Gipec8Eeeu0xXdbba9frFj0=OqFfea0dXdd9vqai=hGuQ8kuc9pgc9s8qqaq=dirpe0xb9q8qiLsFr0=vr0=vr0dc8meaabaqaciaacaGaaeqabaqabeGadaaakeaacqWG0baDcqGGQaGkcqGH9aqpcqGHsislcqaIXaqmcqGHRaWkdaWcaaqaaiabikdaYiabcIcaOiabdsha0jabgkHiTiabdsha0naaBaaaleaacyGGTbqBcqGGPbqAcqGGUbGBaeqaaOGaeiykaKcabaGaemiDaq3aaSbaaSqaaiGbc2gaTjabcggaHjabcIha4bqabaGccqGHsislcqWG0baDdaWgaaWcbaGagiyBa0MaeiyAaKMaeiOBa4gabeaaaaGccqGGSaalaaa@4AFC@

where *t*_min _and *t*_max _are respectively the first and last time points.

Our aim is to model the time-dependent genotypic values for different QTL genotypes *j*_1_*j*_2_, using the orthogonal Lengedre polynomial with a particular order *r*. A family of such polynomials is denoted by

P→r(t*)=[P0(t*),P1(t*),⋯,Pr(t*)]
 MathType@MTEF@5@5@+=feaafiart1ev1aaatCvAUfKttLearuWrP9MDH5MBPbIqV92AaeXatLxBI9gBaebbnrfifHhDYfgasaacH8akY=wiFfYdH8Gipec8Eeeu0xXdbba9frFj0=OqFfea0dXdd9vqai=hGuQ8kuc9pgc9s8qqaq=dirpe0xb9q8qiLsFr0=vr0=vr0dc8meaabaqaciaacaGaaeqabaqabeGadaaakeaacuWGqbaugaWcamaaBaaaleaacqWGYbGCaeqaaOGaeiikaGIaemiDaqNaeiOkaOIaeiykaKIaeyypa0Jaei4waSLaemiuaa1aaSbaaSqaaiabicdaWaqabaGccqGGOaakcqWG0baDcqGGQaGkcqGGPaqkcqGGSaalcqWGqbaudaWgaaWcbaGaeGymaedabeaakiabcIcaOiabdsha0jabcQcaQiabcMcaPiabcYcaSiabl+UimjabcYcaSiabdcfaqnaaBaaaleaacqWGYbGCaeqaaOGaeiikaGIaemiDaqNaeiOkaOIaeiykaKIaeiyxa0faaa@4F02@

and a vector of genotypic values, which is time-independent, denoted by

w→j1j2=(wj1j20,wj1j21,⋯,wj1j2r)′.
 MathType@MTEF@5@5@+=feaafiart1ev1aaatCvAUfKttLearuWrP9MDH5MBPbIqV92AaeXatLxBI9gBaebbnrfifHhDYfgasaacH8akY=wiFfYdH8Gipec8Eeeu0xXdbba9frFj0=OqFfea0dXdd9vqai=hGuQ8kuc9pgc9s8qqaq=dirpe0xb9q8qiLsFr0=vr0=vr0dc8meaabaqaciaacaGaaeqabaqabeGadaaakeaacuWG3bWDgaWcamaaBaaaleaacqWGQbGAdaWgaaadbaGaeGymaedabeaaliabdQgaQnaaBaaameaacqaIYaGmaeqaaaWcbeaakiabg2da9iabcIcaOiabdEha3naaBaaaleaacqWGQbGAdaWgaaadbaGaeGymaedabeaaliabdQgaQnaaBaaameaacqaIYaGmaeqaaSGaeGimaadabeaakiabcYcaSiabdEha3naaBaaaleaacqWGQbGAdaWgaaadbaGaeGymaedabeaaliabdQgaQnaaBaaameaacqaIYaGmaeqaaSGaeGymaedabeaakiabcYcaSiabl+UimjabcYcaSiabdEha3naaBaaaleaacqWGQbGAdaWgaaadbaGaeGymaedabeaaliabdQgaQnaaBaaameaacqaIYaGmaeqaaSGaemOCaihabeaakiqbcMcaPyaafaGaeiOla4caaa@5323@

The time-dependent genotypic values uj1j2
 MathType@MTEF@5@5@+=feaafiart1ev1aaatCvAUfKttLearuWrP9MDH5MBPbIqV92AaeXatLxBI9gBaebbnrfifHhDYfgasaacH8akY=wiFfYdH8Gipec8Eeeu0xXdbba9frFj0=OqFfea0dXdd9vqai=hGuQ8kuc9pgc9s8qqaq=dirpe0xb9q8qiLsFr0=vr0=vr0dc8meaabaqaciaacaGaaeqabaqabeGadaaakeaacqWG1bqDdaWgaaWcbaGaemOAaO2aaSbaaWqaaiabigdaXaqabaWccqWGQbGAdaWgaaadbaGaeGOmaidabeaaaSqabaaaaa@3357@(*t*) can be described as a linear combination of w→j1j2
 MathType@MTEF@5@5@+=feaafiart1ev1aaatCvAUfKttLearuWrP9MDH5MBPbIqV92AaeXatLxBI9gBaebbnrfifHhDYfgasaacH8akY=wiFfYdH8Gipec8Eeeu0xXdbba9frFj0=OqFfea0dXdd9vqai=hGuQ8kuc9pgc9s8qqaq=dirpe0xb9q8qiLsFr0=vr0=vr0dc8meaabaqaciaacaGaaeqabaqabeGadaaakeaacuWG3bWDgaWcamaaBaaaleaacqWGQbGAdaWgaaadbaGaeGymaedabeaaliabdQgaQnaaBaaameaacqaIYaGmaeqaaaWcbeaaaaa@336D@ weighted by the family of the polynomials, i.e.,

uj1j2(t)=P→r(t*)w→j1j2.     (3)
 MathType@MTEF@5@5@+=feaafiart1ev1aaatCvAUfKttLearuWrP9MDH5MBPbIqV92AaeXatLxBI9gBaebbnrfifHhDYfgasaacH8akY=wiFfYdH8Gipec8Eeeu0xXdbba9frFj0=OqFfea0dXdd9vqai=hGuQ8kuc9pgc9s8qqaq=dirpe0xb9q8qiLsFr0=vr0=vr0dc8meaabaqaciaacaGaaeqabaqabeGadaaakeaacqWG1bqDdaWgaaWcbaGaemOAaO2aaSbaaWqaaiabigdaXaqabaWccqWGQbGAdaWgaaadbaGaeGOmaidabeaaaSqabaGccqGGOaakcqWG0baDcqGGPaqkcqGH9aqpcuWGqbaugaWcamaaBaaaleaacqWGYbGCaeqaaOGaeiikaGIaemiDaqNaeiOkaOIaeiykaKIafm4DaCNbaSaadaWgaaWcbaGaemOAaO2aaSbaaWqaaiabigdaXaqabaWccqWGQbGAdaWgaaadbaGaeGOmaidabeaaaSqabaGccqGGUaGlcaWLjaGaaCzcamaabmaabaGaeG4mamdacaGLOaGaayzkaaaaaa@49D7@

Substituting the mean vector of the likelihood (1) by the above expression (3), we will need to estimate time-invariant genotypic values for the longitudinal trait, w→j1j2
 MathType@MTEF@5@5@+=feaafiart1ev1aaatCvAUfKttLearuWrP9MDH5MBPbIqV92AaeXatLxBI9gBaebbnrfifHhDYfgasaacH8akY=wiFfYdH8Gipec8Eeeu0xXdbba9frFj0=OqFfea0dXdd9vqai=hGuQ8kuc9pgc9s8qqaq=dirpe0xb9q8qiLsFr0=vr0=vr0dc8meaabaqaciaacaGaaeqabaqabeGadaaakeaacuWG3bWDgaWcamaaBaaaleaacqWGQbGAdaWgaaadbaGaeGymaedabeaaliabdQgaQnaaBaaameaacqaIYaGmaeqaaaWcbeaaaaa@336D@, and the genotypic mean for the event trait, vj1j2
 MathType@MTEF@5@5@+=feaafiart1ev1aaatCvAUfKttLearuWrP9MDH5MBPbIqV92AaeXatLxBI9gBaebbnrfifHhDYfgasaacH8akY=wiFfYdH8Gipec8Eeeu0xXdbba9frFj0=OqFfea0dXdd9vqai=hGuQ8kuc9pgc9s8qqaq=dirpe0xb9q8qiLsFr0=vr0=vr0dc8meaabaqaciaacaGaaeqabaqabeGadaaakeaacqWG2bGDdaWgaaWcbaGaemOAaO2aaSbaaWqaaiabigdaXaqabaWccqWGQbGAdaWgaaadbaGaeGOmaidabeaaaSqabaaaaa@3359@.

### Modelling the covariance matrix

A general form for the covariance matrix among longitudinal trajectories and development event in the likelihood (1) is expressed as

∑=(∑y∑yz∑zyσz2),     (4)
 MathType@MTEF@5@5@+=feaafiart1ev1aaatCvAUfKttLearuWrP9MDH5MBPbIqV92AaeXatLxBI9gBaebbnrfifHhDYfgasaacH8akY=wiFfYdH8Gipec8Eeeu0xXdbba9frFj0=OqFfea0dXdd9vqai=hGuQ8kuc9pgc9s8qqaq=dirpe0xb9q8qiLsFr0=vr0=vr0dc8meaabaqaciaacaGaaeqabaqabeGadaaakeaacqGHris5cqGH9aqpdaqadaqaauaabeqaciaaaeaacqGHris5daWgaaWcbaGaemyEaKhabeaaaOqaaiabggHiLpaaBaaaleaacqWG5bqEcqWG6bGEaeqaaaGcbaGaeyyeIu+aaSbaaSqaaiabdQha6jabdMha5bqabaaakeaaiiGacqWFdpWCdaqhaaWcbaGaemOEaOhabaGaeGOmaidaaaaaaOGaayjkaiaawMcaaiabcYcaSiaaxMaacaWLjaWaaeWaaeaacqaI0aanaiaawIcacaGLPaaaaaa@46FB@

where Σ_*y *_and σz2
 MathType@MTEF@5@5@+=feaafiart1ev1aaatCvAUfKttLearuWrP9MDH5MBPbIqV92AaeXatLxBI9gBaebbnrfifHhDYfgasaacH8akY=wiFfYdH8Gipec8Eeeu0xXdbba9frFj0=OqFfea0dXdd9vqai=hGuQ8kuc9pgc9s8qqaq=dirpe0xb9q8qiLsFr0=vr0=vr0dc8meaabaqaciaacaGaaeqabaqabeGadaaakeaaiiGacqWFdpWCdaqhaaWcbaGaemOEaOhabaGaeGOmaidaaaaa@3112@ are the covariance matrix and variance for the longitudinal and event traits, respectively, and Σ_*yz *_= ∑′zy
 MathType@MTEF@5@5@+=feaafiart1ev1aaatCvAUfKttLearuWrP9MDH5MBPbIqV92AaeXatLxBI9gBaebbnrfifHhDYfgasaacH8akY=wiFfYdH8Gipec8Eeeu0xXdbba9frFj0=OqFfea0dXdd9vqai=hGuQ8kuc9pgc9s8qqaq=dirpe0xb9q8qiLsFr0=vr0=vr0dc8meaabaqaciaacaGaaeqabaqabeGadaaakeaacuGHris5gaqbamaaBaaaleaacqWG6bGEcqWG5bqEaeqaaaaa@3180@ is the covariance matrix between these two types of traits. The structures of Σ_*y *_and Σ_*yz *_can be empirically modelled on the basis of prior knowledge or results. Several approaches for parametric modelling of the covariance matrix, reviewed by Zimmerman and Nunez-Anton [20], can be utilized.

The most common approach for modelling the covariance structure is based on a variance-correlation specification, in which functions for the responses' variances and correlations are specified. In previous QTL mapping [10-14], the covariance structure for longitudinal traits is modelled by the simplest, most parsimonious and most flexible first-order autoregressive (AR(1)) model in which there are two parameters, stationary variance (σy2
 MathType@MTEF@5@5@+=feaafiart1ev1aaatCvAUfKttLearuWrP9MDH5MBPbIqV92AaeXatLxBI9gBaebbnrfifHhDYfgasaacH8akY=wiFfYdH8Gipec8Eeeu0xXdbba9frFj0=OqFfea0dXdd9vqai=hGuQ8kuc9pgc9s8qqaq=dirpe0xb9q8qiLsFr0=vr0=vr0dc8meaabaqaciaacaGaaeqabaqabeGadaaakeaaiiGacqWFdpWCdaqhaaWcbaGaemyEaKhabaGaeGOmaidaaaaa@3110@) and correlation (*ρ*_*y*_). Relaxing the stationary variance assumption for growth data, Wu et al. [15] adopted a transform-both-sides (TBS) model to obtain an empirically homogeneous variance. The results from simulation studies suggest that the TBS-based mapping model provides more precise estimates for curve parameters and residual variance-correlation than the untrans-formed model.

The TBS-based model displays the potential to relax the assumption of variance stationarity, but the covariance stationarity issue remains unsolved. Zimmerman and Núñez-Antón [20] proposed a so-called structured antedependence (SAD) model to model the age-specific change of correlation in the analysis of longitudinal traits. The SAD model has been employed in several studies and displays many favorable properties [21,22].

The emergence of a developmental event (*z*) at time t* can be correlated with the longitudinal trait. For example, larger tumor sizes may be likely to lead to earlier malignance of cancer than smaller tumor sizes. An AIDS patient would die when his/her HIV load accumulates to a particularly high level. In plants, first flowering only appears after some investment of vegetative growth. All such common knowledge suggests that the correlation between the event trait at time t* and longitudinal trait measured at time *t *(before t*) decays with time difference (t* - *t*). In fact, a similar pattern of correlation should also hold for t > t* because of the autocorrelation nature. With all this consideration, the correlation between the event and longitudinal traits can be modelled by the power equation, expressed as

corr(y(t),z(t*))={{η(t*λ−tλ)/λ+1,λ≠0ηln⁡(t*/t)+1,λ=0,for t*>t,{η(tλ−t*λ)/λ+1,λ≠0ηln⁡(t/t*)+1,λ=0,for t>t*     (5)
 MathType@MTEF@5@5@+=feaafiart1ev1aaatCvAUfKttLearuWrP9MDH5MBPbIqV92AaeXatLxBI9gBaebbnrfifHhDYfgasaacH8akY=wiFfYdH8Gipec8Eeeu0xXdbba9frFj0=OqFfea0dXdd9vqai=hGuQ8kuc9pgc9s8qqaq=dirpe0xb9q8qiLsFr0=vr0=vr0dc8meaabaqaciaacaGaaeqabaqabeGadaaakeaacqqGJbWycqqGVbWBcqqGYbGCcqqGYbGCcqGGOaakcqWG5bqEcqGGOaakcqWG0baDcqGGPaqkcqGGSaalcqWG6bGEcqGGOaakcqWG0baDcqGGQaGkcqGGPaqkcqGGPaqkcqGH9aqpdaGabaqaauaabaqaceaaaeaadaGabaqaauaabaqaciaaaeaaiiGacqWF3oaAdaahaaWcbeqaaiabcIcaOiabdsha0jabcQcaQmaaCaaameqabaGae83UdWgaaSGaeyOeI0IaemiDaq3aaWbaaWqabeaacqWF7oaBaaWccqGGPaqkcqGGVaWlcqWF7oaBcqGHRaWkcqaIXaqmaaGccqGGSaalaeaacqWF7oaBcqGHGjsUcqaIWaamaeaacqWF3oaAdaahaaWcbeqaaiGbcYgaSjabc6gaUjabcIcaOiabdsha0jabcQcaQiabc+caViabdsha0jabcMcaPiabgUcaRiabigdaXaaakiabcYcaSaqaaiab=T7aSjabg2da9iabicdaWiabcYcaSaaaaiaawUhaaiaaxMaacqqGMbGzcqqGVbWBcqqGYbGCcqqGGaaicqWG0baDcqGGQaGkcqGH+aGpcqWG0baDcqGGSaalaeaadaGabaqaauaabaqaciaaaeaacqWF3oaAdaahaaWcbeqaaiabcIcaOiabdsha0naaCaaameqabaGae83UdWgaaSGaeyOeI0IaemiDaqNaeiOkaOYaaWbaaWqabeaacqWF7oaBaaWccqGGPaqkcqGGVaWlcqaH7oaBcqGHRaWkcqaIXaqmaaGccqGGSaalaeaacqWF7oaBcqGHGjsUcqaIWaamaeaacqWF3oaAdaahaaWcbeqaaiGbcYgaSjabc6gaUjabcIcaOiabdsha0jabc+caViabdsha0jabcQcaQiabcMcaPiabgUcaRiabigdaXaaakiabcYcaSaqaaiab=T7aSjabg2da9iabicdaWiabcYcaSaaaaiaawUhaaiaaxMaacqqGMbGzcqqGVbWBcqqGYbGCcqqGGaaicqWG0baDcqGH+aGpcqWG0baDcqGGQaGkaaaacaGL7baacaWLjaGaaCzcamaabmaabaGaeGynaudacaGLOaGaayzkaaaaaa@AC82@

where 0 ≤ *η *≤ 1. Equation (5) suggests that the event is correlated with the longitudinal trait, to the same extent, before and after its emergence. The event trait should be individual-specific when it is the timing of development, such as the time to first flower. In this case, Equation (5) and, therefore, the covariance matrix (4), expressed as Σ_*i*_, should be individual-specific. If Σ_*y *_is modelled by the AR(1) model, one can derive the explicit expressions of the determinant and inverse of Σ_*i *_specified by (σy2
 MathType@MTEF@5@5@+=feaafiart1ev1aaatCvAUfKttLearuWrP9MDH5MBPbIqV92AaeXatLxBI9gBaebbnrfifHhDYfgasaacH8akY=wiFfYdH8Gipec8Eeeu0xXdbba9frFj0=OqFfea0dXdd9vqai=hGuQ8kuc9pgc9s8qqaq=dirpe0xb9q8qiLsFr0=vr0=vr0dc8meaabaqaciaacaGaaeqabaqabeGadaaakeaaiiGacqWFdpWCdaqhaaWcbaGaemyEaKhabaGaeGOmaidaaaaa@3110@, *ρ*_*y*_, σz2
 MathType@MTEF@5@5@+=feaafiart1ev1aaatCvAUfKttLearuWrP9MDH5MBPbIqV92AaeXatLxBI9gBaebbnrfifHhDYfgasaacH8akY=wiFfYdH8Gipec8Eeeu0xXdbba9frFj0=OqFfea0dXdd9vqai=hGuQ8kuc9pgc9s8qqaq=dirpe0xb9q8qiLsFr0=vr0=vr0dc8meaabaqaciaacaGaaeqabaqabeGadaaakeaaiiGacqWFdpWCdaqhaaWcbaGaemOEaOhabaGaeGOmaidaaaaa@3112@, *η*, *λ*).

### Computational algorithms

The unknown parameters (**Ω**) contained with the mixture model (1) include three types, QTL-marker recombination fractions for a pedigree or QTL-marker linkage disequilibria for a natural population reflected in the conditional probabilities ϖj1j2|i
 MathType@MTEF@5@5@+=feaafiart1ev1aaatCvAUfKttLearuWrP9MDH5MBPbIqV92AaeXatLxBI9gBaebbnrfifHhDYfgasaacH8akY=wiFfYdH8Gipec8Eeeu0xXdbba9frFj0=OqFfea0dXdd9vqai=hGuQ8kuc9pgc9s8qqaq=dirpe0xb9q8qiLsFr0=vr0=vr0dc8meaabaqaciaacaGaaeqabaqabeGadaaakeaaiiGacqWFwpGDdaWgaaWcbaGaemOAaO2aaSbaaWqaaiabigdaXaqabaWccqWGQbGAdaWgaaadbaGaeGOmaidabeaaliabcYha8jabdMgaPbqabaaaaa@369F@, the curve parameters (w→j1j2
 MathType@MTEF@5@5@+=feaafiart1ev1aaatCvAUfKttLearuWrP9MDH5MBPbIqV92AaeXatLxBI9gBaebbnrfifHhDYfgasaacH8akY=wiFfYdH8Gipec8Eeeu0xXdbba9frFj0=OqFfea0dXdd9vqai=hGuQ8kuc9pgc9s8qqaq=dirpe0xb9q8qiLsFr0=vr0=vr0dc8meaabaqaciaacaGaaeqabaqabeGadaaakeaacuWG3bWDgaWcamaaBaaaleaacqWGQbGAdaWgaaadbaGaeGymaedabeaaliabdQgaQnaaBaaameaacqaIYaGmaeqaaaWcbeaaaaa@336D@, vj1j2
 MathType@MTEF@5@5@+=feaafiart1ev1aaatCvAUfKttLearuWrP9MDH5MBPbIqV92AaeXatLxBI9gBaebbnrfifHhDYfgasaacH8akY=wiFfYdH8Gipec8Eeeu0xXdbba9frFj0=OqFfea0dXdd9vqai=hGuQ8kuc9pgc9s8qqaq=dirpe0xb9q8qiLsFr0=vr0=vr0dc8meaabaqaciaacaGaaeqabaqabeGadaaakeaacqWG2bGDdaWgaaWcbaGaemOAaO2aaSbaaWqaaiabigdaXaqabaWccqWGQbGAdaWgaaadbaGaeGOmaidabeaaaSqabaaaaa@3359@) that model the mean vector, and the parameters (σy2
 MathType@MTEF@5@5@+=feaafiart1ev1aaatCvAUfKttLearuWrP9MDH5MBPbIqV92AaeXatLxBI9gBaebbnrfifHhDYfgasaacH8akY=wiFfYdH8Gipec8Eeeu0xXdbba9frFj0=OqFfea0dXdd9vqai=hGuQ8kuc9pgc9s8qqaq=dirpe0xb9q8qiLsFr0=vr0=vr0dc8meaabaqaciaacaGaaeqabaqabeGadaaakeaaiiGacqWFdpWCdaqhaaWcbaGaemyEaKhabaGaeGOmaidaaaaa@3110@, *ρ*_*y*_, σz2
 MathType@MTEF@5@5@+=feaafiart1ev1aaatCvAUfKttLearuWrP9MDH5MBPbIqV92AaeXatLxBI9gBaebbnrfifHhDYfgasaacH8akY=wiFfYdH8Gipec8Eeeu0xXdbba9frFj0=OqFfea0dXdd9vqai=hGuQ8kuc9pgc9s8qqaq=dirpe0xb9q8qiLsFr0=vr0=vr0dc8meaabaqaciaacaGaaeqabaqabeGadaaakeaaiiGacqWFdpWCdaqhaaWcbaGaemOEaOhabaGaeGOmaidaaaaa@3112@, *η*, *λ*) that model the structure of the covariance matrix. We derived the EM algorithm to estimate these parameters. Using the (prior) conditional probability and the likelihood, we define the posterior probability for individual *i *to bear on a QTL genotype *j*_1_*j*_2 _as

∏j1j2|i=ϖj1j2|ifj1j2(yi,zi;uj1j2,vj1j2,∑)∑j1=02∑j2=02[ϖj1j2|ifj1j2(yi,zi;uj1j2,vj1j2,∑)],     (6)
 MathType@MTEF@5@5@+=feaafiart1ev1aaatCvAUfKttLearuWrP9MDH5MBPbIqV92AaeXatLxBI9gBaebbnrfifHhDYfgasaacH8akY=wiFfYdH8Gipec8Eeeu0xXdbba9frFj0=OqFfea0dXdd9vqai=hGuQ8kuc9pgc9s8qqaq=dirpe0xb9q8qiLsFr0=vr0=vr0dc8meaabaqaciaacaGaaeqabaqabeGadaaakeaacqGHpis1daWgaaWcbaGaemOAaO2aaSbaaWqaaiabigdaXaqabaWccqWGQbGAdaWgaaadbaGaeGOmaidabeaaliabcYha8jabdMgaPbqabaGccqGH9aqpdaWcaaqaaGGaciab=z9a2naaBaaaleaacqWGQbGAdaWgaaadbaGaeGymaedabeaaliabdQgaQnaaBaaameaacqaIYaGmaeqaaSGaeiiFaWNaemyAaKgabeaakiabdAgaMnaaBaaaleaacqWGQbGAdaWgaaadbaGaeGymaedabeaaliabdQgaQnaaBaaameaacqaIYaGmaeqaaaWcbeaakiabcIcaOGqabiab+Lha5naaBaaaleaacqWGPbqAaeqaaOGaeiilaWIaemOEaO3aaSbaaSqaaiabdMgaPbqabaGccqGG7aWocqGF1bqDdaWgaaWcbaGaemOAaO2aaSbaaWqaaiabigdaXaqabaWccqWGQbGAdaWgaaadbaGaeGOmaidabeaaaSqabaGccqGGSaalcqWG2bGDdaWgaaWcbaGaemOAaO2aaSbaaWqaaiabigdaXaqabaWccqWGQbGAdaWgaaadbaGaeGOmaidabeaaaSqabaGccqGGSaalcqGHris5cqGGPaqkaeaadaaeWaqaamaaqadabaWaamWaaeaacqWFwpGDdaWgaaWcbaGaemOAaO2aaSbaaWqaaiabigdaXaqabaWccqWGQbGAdaWgaaadbaGaeGOmaidabeaaliabcYha8jabdMgaPbqabaGccqWGMbGzdaWgaaWcbaGaemOAaO2aaSbaaWqaaiabigdaXaqabaWccqWGQbGAdaWgaaadbaGaeGOmaidabeaaaSqabaGcdaqadaqaaiab+Lha5naaBaaaleaacqWGPbqAaeqaaOGaeiilaWIaemOEaO3aaSbaaSqaaiabdMgaPbqabaGccqGG7aWocqGF1bqDdaWgaaWcbaGaemOAaO2aaSbaaWqaaiabigdaXaqabaWccqWGQbGAdaWgaaadbaGaeGOmaidabeaaaSqabaGccqGGSaalcqWG2bGDdaWgaaWcbaGaemOAaO2aaSbaaWqaaiabigdaXaqabaWccqWGQbGAdaWgaaadbaGaeGOmaidabeaaaSqabaGccqGGSaalcqGHris5aiaawIcacaGLPaaaaiaawUfacaGLDbaaaSqaaiabdQgaQnaaBaaameaacqaIYaGmaeqaaSGaeyypa0JaeGimaadabaGaeGOmaidaniabggHiLdaaleaacqWGQbGAdaWgaaadbaGaeGymaedabeaaliabg2da9iabicdaWaqaaiabikdaYaqdcqGHris5aaaakiabcYcaSiaaxMaacaWLjaWaaeWaaeaacqaI2aGnaiaawIcacaGLPaaaaaa@A2CD@

The posterior probabilities are then used to derive a closed-form maximum likelihood estimates of the QTL locations, expressed as the ratio of recombination fractions, for linkage analysis or QTL-marker haplotype frequencies for linkage disequilibrium analysis [17]. For functional mapping, in which the mean vectors and covariance matrix are modelled by mathematical parameters based on non-linear equations, it is impossible to derive the closed forms for these parameters, the simplex algorithm, widely used in operations research, is found to provide a fast and precise estimation of the curve parameters and the parameters that model the residual covariances [23]. Thus, we implement the simplex algorithm in the maximization process of the EM algorithm.

For linkage analysis based on an experimental cross, ϖj1j2|i
 MathType@MTEF@5@5@+=feaafiart1ev1aaatCvAUfKttLearuWrP9MDH5MBPbIqV92AaeXatLxBI9gBaebbnrfifHhDYfgasaacH8akY=wiFfYdH8Gipec8Eeeu0xXdbba9frFj0=OqFfea0dXdd9vqai=hGuQ8kuc9pgc9s8qqaq=dirpe0xb9q8qiLsFr0=vr0=vr0dc8meaabaqaciaacaGaaeqabaqabeGadaaakeaaiiGacqWFwpGDdaWgaaWcbaGaemOAaO2aaSbaaWqaaiabigdaXaqabaWccqWGQbGAdaWgaaadbaGaeGOmaidabeaaliabcYha8jabdMgaPbqabaaaaa@369F@'s are expressed in the recombination fraction between the QTL and two flanking markers. In practical computations, the QTL position parameter can be viewed as a fixed parameter because a putative QTL can be searched at every 1 or 2 cM on a map interval bracketed by two markers throughout the entire genome. The amount of support for a QTL at a particular map position is often displayed graphically through the use of likelihood maps or profiles, which plot the likelihood ratio test statistic as a function of map position of the putative QTL. The peak of the profile corresponds to the position of the QTL over the genome.

For linkage disequilibrium analysis of a natural population, we have derived a closed form for the EM algorithm to estimate QTL-marker haplotype frequencies. From the estimated haplotype frequencies, the allele frequencies of QTL and QTL-marker linkage disequilibria can be estimated. How the markers are associated with the underlying QTL in the population can be tested for the significance of QTL-marker linkage disequilibria.

After the point estimates of parameters are obtained by the EM algorithm, the approximate variance-covariance matrix and the sampling errors of the estimates (Ω^
 MathType@MTEF@5@5@+=feaafiart1ev1aaatCvAUfKttLearuWrP9MDH5MBPbIqV92AaeXatLxBI9gBaebbnrfifHhDYfgasaacH8akY=wiFfYdH8Gipec8Eeeu0xXdbba9frFj0=OqFfea0dXdd9vqai=hGuQ8kuc9pgc9s8qqaq=dirpe0xb9q8qiLsFr0=vr0=vr0dc8meaabaqaciaacaGaaeqabaqabeGadaaakeaaiiqacuWFPoWvgaqcaaaa@2E4F@) can be estimated. The techniques for so doing involve calculation of the incomplete-data information matrix which is the negative second-order derivative of the incomplete-data log-likelihood. The incomplete-data information can be calculated by extracting the information for the missing data from the information for the complete data [24].

### Order selection

For a QTL to be detected, we need to determine the optimal order for the Legendre polynomial that fits the data. We propose using the AIC information criterion to select the best model. The AIC value at a particular order, *r*, is calculated by

AIC = -2 ln *L*(Ω^
 MathType@MTEF@5@5@+=feaafiart1ev1aaatCvAUfKttLearuWrP9MDH5MBPbIqV92AaeXatLxBI9gBaebbnrfifHhDYfgasaacH8akY=wiFfYdH8Gipec8Eeeu0xXdbba9frFj0=OqFfea0dXdd9vqai=hGuQ8kuc9pgc9s8qqaq=dirpe0xb9q8qiLsFr0=vr0=vr0dc8meaabaqaciaacaGaaeqabaqabeGadaaakeaaiiqacuWFPoWvgaqcaaaa@2E4F@|*r*) + 2 dimension(**Ω**|*r*),     (7)

where (Ω^
 MathType@MTEF@5@5@+=feaafiart1ev1aaatCvAUfKttLearuWrP9MDH5MBPbIqV92AaeXatLxBI9gBaebbnrfifHhDYfgasaacH8akY=wiFfYdH8Gipec8Eeeu0xXdbba9frFj0=OqFfea0dXdd9vqai=hGuQ8kuc9pgc9s8qqaq=dirpe0xb9q8qiLsFr0=vr0=vr0dc8meaabaqaciaacaGaaeqabaqabeGadaaakeaaiiqacuWFPoWvgaqcaaaa@2E4F@|*r*) is the the MLE of parameters for the Legendre polynomial of order *r *and dimension (**Ω**|*r*) represents the number of independent parameters under order *r*.

Also, Bayesian Information Criterion (BIC) [25] is used to determine the optimal order of the Legendre function, which is calculated by

BIC = -2 ln *L*(Ω^
 MathType@MTEF@5@5@+=feaafiart1ev1aaatCvAUfKttLearuWrP9MDH5MBPbIqV92AaeXatLxBI9gBaebbnrfifHhDYfgasaacH8akY=wiFfYdH8Gipec8Eeeu0xXdbba9frFj0=OqFfea0dXdd9vqai=hGuQ8kuc9pgc9s8qqaq=dirpe0xb9q8qiLsFr0=vr0=vr0dc8meaabaqaciaacaGaaeqabaqabeGadaaakeaaiiqacuWFPoWvgaqcaaaa@2E4F@|*r*) + 2 dimension(**Ω**|*r*) ln (*nT*).     (8)

As compared to AIC, BIC adjusts the effects of sample size and the number of time points measured.

### Hypothesis tests

Our model allows for a number of hypothesis tests to examine the genetic control of growth processes [14]. All these tests are helpful to address biological questions related to the genetic control mechanisms of growth. Testing whether specific QTL exist to affect the longitudinal and event processes is a first step toward the understanding of the detailed genetic architecture of complex phenotypes. This can be tested by formulating the following hypotheses,

*H*_0 _: uj1j2
 MathType@MTEF@5@5@+=feaafiart1ev1aaatCvAUfKttLearuWrP9MDH5MBPbIqV92AaeXatLxBI9gBaebbnrfifHhDYfgasaacH8akY=wiFfYdH8Gipec8Eeeu0xXdbba9frFj0=OqFfea0dXdd9vqai=hGuQ8kuc9pgc9s8qqaq=dirpe0xb9q8qiLsFr0=vr0=vr0dc8meaabaqaciaacaGaaeqabaqabeGadaaakeaaieqacqWF1bqDdaWgaaWcbaGaemOAaO2aaSbaaWqaaiabigdaXaqabaWccqWGQbGAdaWgaaadbaGaeGOmaidabeaaaSqabaaaaa@335D@ = **u **and vj1j2
 MathType@MTEF@5@5@+=feaafiart1ev1aaatCvAUfKttLearuWrP9MDH5MBPbIqV92AaeXatLxBI9gBaebbnrfifHhDYfgasaacH8akY=wiFfYdH8Gipec8Eeeu0xXdbba9frFj0=OqFfea0dXdd9vqai=hGuQ8kuc9pgc9s8qqaq=dirpe0xb9q8qiLsFr0=vr0=vr0dc8meaabaqaciaacaGaaeqabaqabeGadaaakeaacqWG2bGDdaWgaaWcbaGaemOAaO2aaSbaaWqaaiabigdaXaqabaWccqWGQbGAdaWgaaadbaGaeGOmaidabeaaaSqabaaaaa@3359@ = *v *vs. *H*_1_: Not all equalities in *H*_0 _hold.     (9)

The *H*_0 _states that there is no QTL affecting longitudinal and event processes (the reduced model), whereas the *H*_1 _proposes that such a QTL does exist (the full model). The test statistic for testing the hypotheses is calculated as the log-likelihood ratio of the reduced to the full model:

*LR *= -2[ln *L*_0_(Ω˜
 MathType@MTEF@5@5@+=feaafiart1ev1aaatCvAUfKttLearuWrP9MDH5MBPbIqV92AaeXatLxBI9gBaebbnrfifHhDYfgasaacH8akY=wiFfYdH8Gipec8Eeeu0xXdbba9frFj0=OqFfea0dXdd9vqai=hGuQ8kuc9pgc9s8qqaq=dirpe0xb9q8qiLsFr0=vr0=vr0dc8meaabaqaciaacaGaaeqabaqabeGadaaakeaaiiqacuWFPoWvgaacaaaa@2E4E@|**y**, *z*) - ln *L*_1 _(Ω^
 MathType@MTEF@5@5@+=feaafiart1ev1aaatCvAUfKttLearuWrP9MDH5MBPbIqV92AaeXatLxBI9gBaebbnrfifHhDYfgasaacH8akY=wiFfYdH8Gipec8Eeeu0xXdbba9frFj0=OqFfea0dXdd9vqai=hGuQ8kuc9pgc9s8qqaq=dirpe0xb9q8qiLsFr0=vr0=vr0dc8meaabaqaciaacaGaaeqabaqabeGadaaakeaaiiqacuWFPoWvgaqcaaaa@2E4F@|**y**, *z*, **M**)],     (10)

where Ω˜
 MathType@MTEF@5@5@+=feaafiart1ev1aaatCvAUfKttLearuWrP9MDH5MBPbIqV92AaeXatLxBI9gBaebbnrfifHhDYfgasaacH8akY=wiFfYdH8Gipec8Eeeu0xXdbba9frFj0=OqFfea0dXdd9vqai=hGuQ8kuc9pgc9s8qqaq=dirpe0xb9q8qiLsFr0=vr0=vr0dc8meaabaqaciaacaGaaeqabaqabeGadaaakeaaiiqacuWFPoWvgaacaaaa@2E4E@ and Ω^
 MathType@MTEF@5@5@+=feaafiart1ev1aaatCvAUfKttLearuWrP9MDH5MBPbIqV92AaeXatLxBI9gBaebbnrfifHhDYfgasaacH8akY=wiFfYdH8Gipec8Eeeu0xXdbba9frFj0=OqFfea0dXdd9vqai=hGuQ8kuc9pgc9s8qqaq=dirpe0xb9q8qiLsFr0=vr0=vr0dc8meaabaqaciaacaGaaeqabaqabeGadaaakeaaiiqacuWFPoWvgaqcaaaa@2E4F@ denote the MLEs of the unknown parameters under *H*_0 _and *H*_1_, respectively. Because the *LR *calculated by equation (9) may not be asymptotically *χ*^2^-distributed with eleven degrees of freedom due to violation of regularity conditions, an empirical approach for determining the critical threshold based on permutation tests is used. By repeatedly shuffling the relationships between marker genotypes and phenotypes, a series of the maximum log-likelihood ratios are calculated, from the distribution of which the critical threshold is determined.

After the QTL are detected to be significant for both longitudinal and event traits, we need to test whether the detected QTL are significant separately for each trait. We assume two different genetic settings:

(1) Longitudinal and event traits are under control of the same QTL;

(2) Each process is controlled by different QTL that are linked on the same chromosomal region.

For the first setting, only after it is significant in two separate tests for longitudinal and event traits can the tested QTL be thought to be pleiotropic in affecting both types of traits. For the second setting, we assume that two tested QTL are located in the same interval bracketed by two markers. The comparison of the first and second setting can examine how the detected QTL jointly affect the differentiation in longitudinal and event traits. First, we can test how two genetic mechanisms, pleiotropy or close linkage, contribute to the correlation between these two types of traits. If the two QTL are detected to be significant for both, we then test whether such a correlation is due to pleiotropy or close linkage. Second, when two QTL exist, we can test how they epistatically interact to affect longitudinal trajectories and developmental events. Wu et al. [14] formulated a procedure for testing the epistatic effects on developmental trajectories.

## Results

The proposed joint model is used to analyze growth trajectories and flowering behavior in a forest tree. The study material used was derived from the interspecific hybridization of *Populus *(poplar), *P. deltoides *and *P. eummericana*. This hybrid population was planted at a spacing of 4 × 5 m in the complete randomized design in a field trial near Xuzhou City, Jiangsu Province, China. The total stem heights and diameters measured at the end of each of the first 11 growing seasons are used to calculate stem volume indices (**y**) for QTL analysis. Because the vegetative and reproductive growth processes are generally correlated in plants [26], the ages to first flower (*z*) was predicted by a regression equation for each of these hybrids. Two genetic linkage maps each based on a different parent were constructed for a subset of hybrids (90) with different types of molecular markers that are segregating in a pattern of pseudo-test backcross [27]. Our analysis here will be based on *P. deltoides *(D)-specific linkage map.

**Figure 1:**
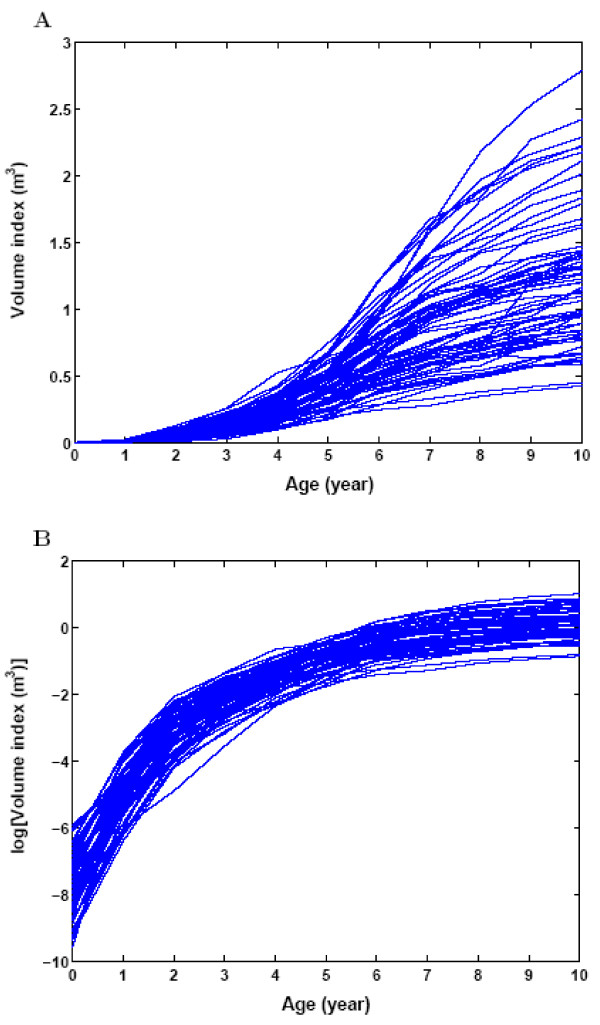
Plots of stem volume index growth vs. ages for each of the 90 genotypes used to construct linkage maps in poplar hybrids (Yin et al. 2002). The relationships between growth and age are displayed for untransformed (**A**) and log-transformed data (**B**).

**Figure 2:**
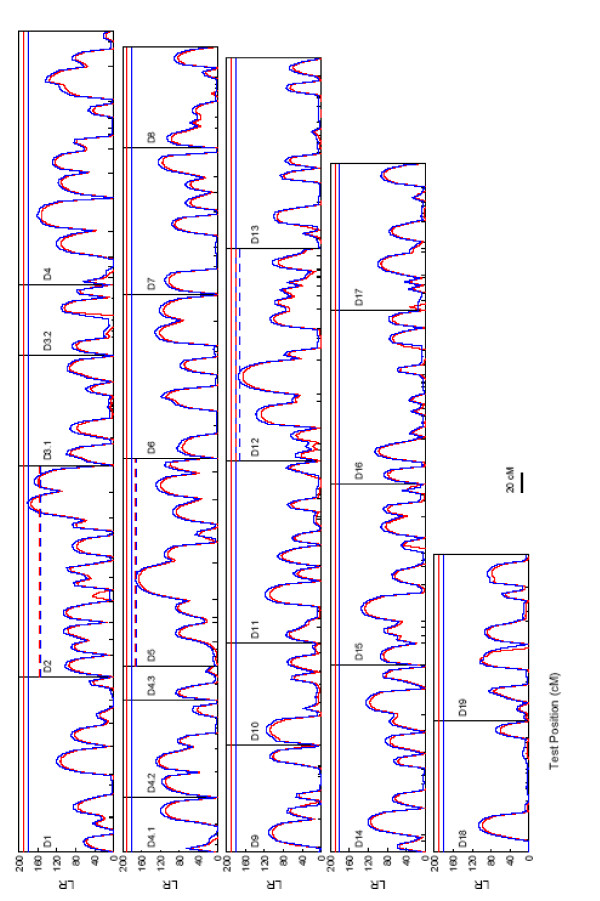
The profile of the log-likelihood ratios between the full (there is a QTL) and reduced (there is no QTL) model that combines stem volume index growth trajectories and flower timing across linkage groups in the *Populus deltoides *parent map. The genomic positions corresponding to the peaks of the curve are the MLEs of the QTL localization. The threshold values for claiming the existence of QTL are given as the horizonal solid lines for the genome-wide level and broken lines for the chromosome-wide level. Blue color corresponds to the unifying model for jointly mapping growth trajectories and flower trait, whereas red color corresponds to a model for mapping growth trajectories only. The positions of markers on the linkage groups (Yin et al. 2002) are indicated at ticks.

Although stem height and diameter for each tree follows a logistic curve [10], the stem volume index derived from these two traits cannot be fit by the growth equation mainly because stem volume has not yet reached its asymptotic growth during this measurement period (Fig. 1A). As shown by Figure 1, the variance of the stem volume index increases markedly with age, but the log-transformation of these indices leads to much parallel curves (Fig. 2B), suggesting that the variance stationarity assumption may be met after the transformation.

We implemented the Legendre function to model the QTL genotypic mean vector of growth trajectories and the TBS-based AR(1) model to approximate the structure of the covariance matrix. The joint model also allows the estimation of the genotypic means and residual variance for the age to first flower, as well as the correlation of this trait with stem wood growth trajectories. Equation (5) provides a general equation for modelling the correlation between the event and longitudinal traits measured at different ages. In this example, it was observed that there were significant correlations between the age to first flower and volume growth at all different ages (-0.29 – -0.82). Thus, for simplicity of computation, we assume that such correlations are consistent across the ages of volume index, denoted as *η*. Using the adjusted ages, we calculated the coefficients of the Legendre polynomials for the first seven orders (Table 1). These coefficients are used to estimate time-dependent genotypic values. The AIC and BIC values calculated consistently suggested an optimal order of 5 to fit stem volume growth (Table 2).

While our joint model was derived to detect two QTL at a time, it was reduced to a one-QTL model because of a limited sample size for sufficient estimates of two-QTL model parameters. For the pseudo-test backcross there are two genotypes, *Qq *(*j *= 1) and *qq *(*j *= 0), at each QTL. Figure 2 illustrates the profile of the log-likelihood ratio (*LR*) values for testing the existence of QTL that control either overall growth curves of stem volume indices from age 0 to 10 years or the ages to first flower, or both, across all of the 19 D-specific linkage groups. We performed 100 permutation tests to determine critical threshold values for declaring the existence of QTL. By comparing the peaks of the *LR *profile with the thresholds, three significant QTL were detected, one on linkage group 2 at the 5% genome-wide testing level and two on linkage groups 5 and 12 at the 5% chromosome-wide testing level (Fig. 2; Table 3). We indicated the positions of these QTL on linkage groups, which correspond to the peaks of the *LR *profile. If only the stem volume growth is analyzed using traditional functional mapping [10], only the QTL on linkage group 2 is detected, suggesting that the joint model displays better power than a single-trait analysis.

Each of the three QTL was tested for their pleiotropic effect on both vegetative growth and reproduction by formulating two independent null hypotheses, one being that the QTL does not affect stem growth and the second being that the QTL does not affect flowering age. The rejection of both the null hypotheses implies that a QTL has a pleiotropic effect on growth and reproduction. As indicated by Table 3, all the detected QTL on linkage groups 2, 5 and 12 only trigger a significant effect on stem volume growth, but neither has an effect on both growth and reproduction.

**Figure 3:**
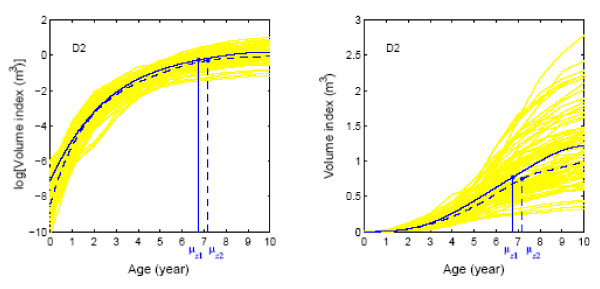
Volume growth curves for two different QTL genotypes for the QTL detected on linkage group 2 by the Legendre polynomial-based model. Left panel: log-transformed curves; Right panel: ante-transformed curves. Growth trajectories for all the individuals studied are indicated in yellow background. The effect of the detected QTL on the time to first flower is indicated.

The MLEs of growth parameters for stem volume indices, covariance-structuring parameters and the parameters dealing with reproductive behaviors, as well as their standard errors estimated from the Fisher information matrix, were tabulated in Table 3. It can be seen that the estimates of all the parameters from our joint model provide reasonable precision, using the estimates of growth curves, we draw two different curves each corresponding to a genotype at each of the detected QTL (Fig. 3). Note that growth curves were first drawn from the estimates of the Legendre parameters (the left panel of Fig. 3) and then transformed back to the normal scale (the right panel of Fig. 3). In general. these QTL are switched on to affect the overall stem growth process after age 4–5 years at which strong inter-tree competition sets in the stand due to canopy closure. Figure 3 also displays genotypic differences in the age to first flower at each of the growth QTL. But as tested, only QTL on linkage group 12 has a significant impact on the age to first flower (Table 3). At this QTL, the slower-growing genotype flowers about 0.7 year earlier than the faster-growing genotype. Through this QTL, the fast-growing attribute and the capacity to efficiently occupy growth resources can be transmitted to the next generation.

## Discussion

A theoretical framework has been constructed for functional mapping of quantitative trait loci (QTL) underlying longitudinal growth [10-15]. Functional mapping was grounded on biological reality that every organism follows universal growth laws that can be derived from fundamental principles for the allocation of metabolic energy between maintenance of existing tissue and the production of new biomass [17]. In a couple with linkage disequilibrium mapping, functional mapping has been extended to map host QTL for HIV dynamics for a natural human population [16].

Although functional mapping has proven to be both biologically and statistically advantageous in terms of the estimates of the QTL positions and effects, its practical applications may be limited for two reasons. First, a longitudinal variable, such as HIV dynamics, tumor growth or plant vegetative growth, may be related to time-to-events, like time to onset of AIDS symptoms, time to first malignancy or time to first flower, through a common set of QTL [28,29]. Second, not all longitudinal data measured at a series of discrete time points can be fit by a mathematical function with biological means.

In this article, we have proposed a joint model for functional mapping of longitudinal trajectories and time-to-events with the nonparametric context. Several statistical models have been proposed to jointly analyze longitudinal and event processes [1-9]. Different from those traditional models, our joint model has been constructed within the mixture model framework, with each mixture component assigned by biological rationale. We incorporated Legendre polynomials to characterize an arbitrary form of growth curves. In a real example for a forest tree, the model has detected a few QTL that affect growth processes and the age to first flower. The detection of the common genetic basis for vegetative and reproductive growth supports the views that any developmental event is not isolated from the growth process [28,29]. Our model provides a complete genetic analysis of growth courses for various organisms at different organization levels. From a statistical perspective, it increases the power of QTL detection and the precision of parameter estimation because the information about growth and development is jointly utilized. Meanwhile, our model allows for the test of several important hypotheses regarding the genetic control of developmental events occurring from fertilized ovum to reproductive maturity.

Our model is based on nonparametric Legendre orthogonal polynomial approaches for growth and development processes. Orthogonal polynomials (including Legendre) have been extensively used in random regression analyses for longitudinal traits with repeated records [18,30-32]. There are several favorable properties for Legendre polynomials to be utilized in curve fitting, i.e., (1) the functions are orthogonal, (2) it is flexible to fit sparse data, (3) higher orders are estimable for high levels of curve complexity and (4) computation is fast because of good convergence. Nonparametric regression methods for modelling the mean structure of longitudinal data have been based on more commonly used B-spline basis functions [33]. Brown et al. [9] extended the B-spline basis to model multiple longitudinal variables. As compared to the B-spline approach that constructs curves from pieces of lower degree polynomials smoothed at selected pointed (knots), Legendre polynomials are simpler in which only fewer regression coefficients are needed to model the curve. However, polynomials often overemphasize the observations at the extremes and may be problematic for high orders of fit due to oscillations at the extremes of the curve [34]. It is therefore worthwhile implementing more flexible B-spline basis functions into the nonparametric functional mapping model.

In our joint model, we assumed that the time-to-event is multivariate normally distributed together with longitudinal data (see also [35]). An alternative to model the distribution of longitudinal and event data is to take the product of the normal distribution function of longitudinal trajectories and the distribution of the event trait and sensoring indicator given the trajectory function [9,36]. In addition, our model should be extended to consider multiple longitudinal variables based on a framework by Lin et al. [5], multiple time-to-events [8] and structured covariance matrices among unbalanced repeated-measures [37]. In order to unravel the genetic architecture of complex phenotypes that are characterized by a network of biological processes, such extensions will be essential. With appropriate improvements, our joint model will have great power to unlock the genetic secrets hidden in various complicated and biologically realistic life processes.

## Conclusion

We have developed a joint statistical model that can detect specific QTL governing both longitudinal traits and developmental processes through either pleiotropic effects or close linkage, or both. This model was integrated by nonparametric approaches that do not rely on mathematical equations to model growth curves. The model will have great implications for integrating longitudinal and event data to gain better insights into comprehensive biology and biomedicine.

## Authors' contributions

ML derived the models, programmed the method and performed data analyses. RW conceived the idea and drafted the manuscript.

## Figures and Tables

**Figure 4:**
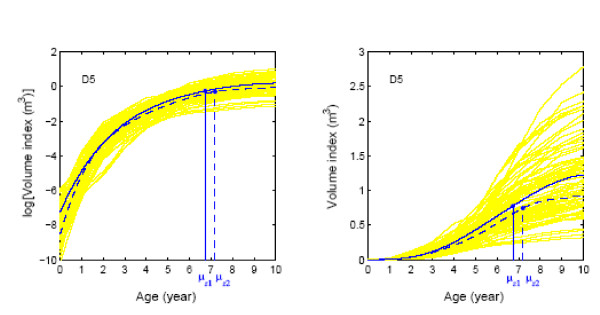
Volume growth curves for two different QTL genotypes for the QTL detected on linkage group 5 by the Legendre polynomial-based model. See Figure 3 for all the explanations.

**Figure 5:**
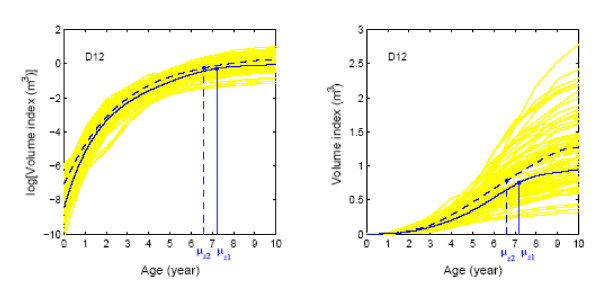
Volume growth curves for two different QTL genotypes for the QTL detected on linkage group 12 by the Legendre polynomial-based model. See Figure 3 for all the explanations.

**Table 1:** Coefficients of the first five Legendre polynomials for adjusted time points (t*) used in the poplar growth study.

*t*	0	1	2	3	4	5	6	7	8	9	10
t*	-1	-4/5	-3/5	-2/5	-1/5	0	1/5	2/5	3/5	4/5	1
*P*_0_(t*)	1	1	1	1	1	1	1	1	1	1	1
*P*_1_(t*)	-1	-4/5	-3/5	-2/5	-1/5	0	1/5	2/5	3/5	4/5	1
*P*_2_(t*)	1	23/50	1/25	-13/50	-11/25	-1/2	-11/25	-13/50	1/25	23/50	1
*P*_3_(t*)	-1	-2/25	9/25	11/25	7/25	0	-7/25	-11/25	-9/25	2/25	1
*P*_4_(t*)	1	-24/103	-51/125	-93/823	29/125	3/8	29/125	-93/823	-51/125	-24/103	1
*P*_5_(t*)	-1	167/418	107/701	-59/218	-274/891	0	274/891	59/218	-107/701	-167/418	1
*P*_6_(t*)	1	-172/439	132/767	163/557	-56/695	-5/16	-56/695	163/557	132/767	-172/439	1
*P*_7_(t*)	-1	110/459	-10/31	13/891	231/787	0	-231/787	-13/891	10/31	-110/459	1

**Table 2:** The AIC and BIC values used to determine the optimal order for the Lengendre polynomials.

Order	AIC	BIC
1	711.0	739.2
2	-255.7	-222.8
3	-802.2	-764.6
4	-959.9	-917.6
5	-983.2	-936.2
6	-985.5	-933.8
7	-1010.3	-931.9

**Table 3:** The MLEs and their sampling errors (SE, in the parentheses) of the QTL position, time-invariant QTL effects on growth curves (expressed in the Legendre polynomials), QTL effect on the time to first flower, residual variance and residual correlation under the log-transformed model for the interspecific poplar hybrid mapping population.

Test/Parameter	**Linkage group 2**		**Linkage group 5**		**Linkage group 12**	
			
	*Qq*	*qq*	*Qq*	*qq*	*Qq*	*qq*
*LR*	186		176		181	
*LR*_*y*_	182		176		176	
*LR*_*z*_	2.7		1.3		2.2	
						
Location	190		96		12	
u^ MathType@MTEF@5@5@+=feaafiart1ev1aaatCvAUfKttLearuWrP9MDH5MBPbIqV92AaeXatLxBI9gBaebbnrfifHhDYfgasaacH8akY=wiFfYdH8Gipec8Eeeu0xXdbba9frFj0=OqFfea0dXdd9vqai=hGuQ8kuc9pgc9s8qqaq=dirpe0xb9q8qiLsFr0=vr0=vr0dc8meaabaqaciaacaGaaeqabaqabeGadaaakeaacuWG1bqDgaqcaaaa@2E2F@_*j*0_	-1.60 (0.0750)	-1.83 (0.0731)	-1.62 (0.0727)	-1.84 (0.0869)	-1.85 (0.0664)	-1.54 (0.0707)
u^ MathType@MTEF@5@5@+=feaafiart1ev1aaatCvAUfKttLearuWrP9MDH5MBPbIqV92AaeXatLxBI9gBaebbnrfifHhDYfgasaacH8akY=wiFfYdH8Gipec8Eeeu0xXdbba9frFj0=OqFfea0dXdd9vqai=hGuQ8kuc9pgc9s8qqaq=dirpe0xb9q8qiLsFr0=vr0=vr0dc8meaabaqaciaacaGaaeqabaqabeGadaaakeaacuWG1bqDgaqcaaaa@2E2F@_*j*1_	3.04 (0.0610)	3.15 (0.0593)	3.09 (0.0622)	3.16 (0.0734)	3.19 (0.0591)	3.02 (0.0626)
u^ MathType@MTEF@5@5@+=feaafiart1ev1aaatCvAUfKttLearuWrP9MDH5MBPbIqV92AaeXatLxBI9gBaebbnrfifHhDYfgasaacH8akY=wiFfYdH8Gipec8Eeeu0xXdbba9frFj0=OqFfea0dXdd9vqai=hGuQ8kuc9pgc9s8qqaq=dirpe0xb9q8qiLsFr0=vr0=vr0dc8meaabaqaciaacaGaaeqabaqabeGadaaakeaacuWG1bqDgaqcaaaa@2E2F@_*j*2_	-1.68 (0.0475)	-1.94 (0.0470)	-1.71 (0.0467)	-1.94 (0.0557)	-1.92 (0.0457)	-1.69 (0.0492)
u^ MathType@MTEF@5@5@+=feaafiart1ev1aaatCvAUfKttLearuWrP9MDH5MBPbIqV92AaeXatLxBI9gBaebbnrfifHhDYfgasaacH8akY=wiFfYdH8Gipec8Eeeu0xXdbba9frFj0=OqFfea0dXdd9vqai=hGuQ8kuc9pgc9s8qqaq=dirpe0xb9q8qiLsFr0=vr0=vr0dc8meaabaqaciaacaGaaeqabaqabeGadaaakeaacuWG1bqDgaqcaaaa@2E2F@_*j*3_	0.60 (0.0403)	0.84 (0.0402)	0.61 (0.0401)	0.85 (0.0475)	0.81 (0.0390)	0.62 (0.0422)
u^ MathType@MTEF@5@5@+=feaafiart1ev1aaatCvAUfKttLearuWrP9MDH5MBPbIqV92AaeXatLxBI9gBaebbnrfifHhDYfgasaacH8akY=wiFfYdH8Gipec8Eeeu0xXdbba9frFj0=OqFfea0dXdd9vqai=hGuQ8kuc9pgc9s8qqaq=dirpe0xb9q8qiLsFr0=vr0=vr0dc8meaabaqaciaacaGaaeqabaqabeGadaaakeaacuWG1bqDgaqcaaaa@2E2F@_*j*4_	-0.17 (0.0295)	-0.45 (0.0279)	-0.18 (0.0305)	-0.48 (0.0335)	-0.44 (0.0283)	-0.16 (0.0306)
u^ MathType@MTEF@5@5@+=feaafiart1ev1aaatCvAUfKttLearuWrP9MDH5MBPbIqV92AaeXatLxBI9gBaebbnrfifHhDYfgasaacH8akY=wiFfYdH8Gipec8Eeeu0xXdbba9frFj0=OqFfea0dXdd9vqai=hGuQ8kuc9pgc9s8qqaq=dirpe0xb9q8qiLsFr0=vr0=vr0dc8meaabaqaciaacaGaaeqabaqabeGadaaakeaacuWG1bqDgaqcaaaa@2E2F@_*j*5_	0.00 (0.0284)	0.21 (0.0270)	0.03 (0.0286)	0.23 (0.0315)	0.21 (0.0274)	-0.01 (0.0292)
σ^y2 MathType@MTEF@5@5@+=feaafiart1ev1aaatCvAUfKttLearuWrP9MDH5MBPbIqV92AaeXatLxBI9gBaebbnrfifHhDYfgasaacH8akY=wiFfYdH8Gipec8Eeeu0xXdbba9frFj0=OqFfea0dXdd9vqai=hGuQ8kuc9pgc9s8qqaq=dirpe0xb9q8qiLsFr0=vr0=vr0dc8meaabaqaciaacaGaaeqabaqabeGadaaakeaaiiGacuWFdpWCgaqcamaaDaaaleaacqWG5bqEaeaacqaIYaGmaaaaaa@3120@	0.28 (0.0339)		0.28 (0.0375)		0.25 (0.0274)	
ρ^ MathType@MTEF@5@5@+=feaafiart1ev1aaatCvAUfKttLearuWrP9MDH5MBPbIqV92AaeXatLxBI9gBaebbnrfifHhDYfgasaacH8akY=wiFfYdH8Gipec8Eeeu0xXdbba9frFj0=OqFfea0dXdd9vqai=hGuQ8kuc9pgc9s8qqaq=dirpe0xb9q8qiLsFr0=vr0=vr0dc8meaabaqaciaacaGaaeqabaqabeGadaaakeaaiiGacuWFbpGCgaqcaaaa@2E83@_*y*_	0.88 (0.0154)		0.87 (0.0176)		0.86 (0.0168)	
v^ MathType@MTEF@5@5@+=feaafiart1ev1aaatCvAUfKttLearuWrP9MDH5MBPbIqV92AaeXatLxBI9gBaebbnrfifHhDYfgasaacH8akY=wiFfYdH8Gipec8Eeeu0xXdbba9frFj0=OqFfea0dXdd9vqai=hGuQ8kuc9pgc9s8qqaq=dirpe0xb9q8qiLsFr0=vr0=vr0dc8meaabaqaciaacaGaaeqabaqabeGadaaakeaacuWG2bGDgaqcaaaa@2E31@_*j*_	6.6 (0.2015)	6.8 (0.1951)	7.1 (0.2200)	7.1 (0.1974)	7.1 (0.2275)	6.7 (0.1813)
σ^z2 MathType@MTEF@5@5@+=feaafiart1ev1aaatCvAUfKttLearuWrP9MDH5MBPbIqV92AaeXatLxBI9gBaebbnrfifHhDYfgasaacH8akY=wiFfYdH8Gipec8Eeeu0xXdbba9frFj0=OqFfea0dXdd9vqai=hGuQ8kuc9pgc9s8qqaq=dirpe0xb9q8qiLsFr0=vr0=vr0dc8meaabaqaciaacaGaaeqabaqabeGadaaakeaaiiGacuWFdpWCgaqcamaaDaaaleaacqWG6bGEaeaacqaIYaGmaaaaaa@3122@	1.42 (0.2394)		1.41 (0.2352)		1.28 (0.1841)	
η^ MathType@MTEF@5@5@+=feaafiart1ev1aaatCvAUfKttLearuWrP9MDH5MBPbIqV92AaeXatLxBI9gBaebbnrfifHhDYfgasaacH8akY=wiFfYdH8Gipec8Eeeu0xXdbba9frFj0=OqFfea0dXdd9vqai=hGuQ8kuc9pgc9s8qqaq=dirpe0xb9q8qiLsFr0=vr0=vr0dc8meaabaqaciaacaGaaeqabaqabeGadaaakeaaiiGacuWF3oaAgaqcaaaa@2E6F@	-0.50 (0.0686)		-0.51 (0.0580)		-0.48 (0.0503)	

The *LR*, *LR*_*y *_and *LR*_*z *_values are the test statistics for testing the existence of a QTL for both growth and the time to first flower, the existence of a QTL for growth but not for the time to first flower, and the existence of a QTL for the time to first flower but not for growth. The locations of the detected QTL are described by the genetic distance (in cM) from the first marker of a linkage group.
